# Evaluation of remote radiologist-interpreted point-of-care ultrasound for suspected dengue patients in a primary health care facility in Colombia

**DOI:** 10.1186/s40249-023-01141-9

**Published:** 2023-09-28

**Authors:** Lyda Osorio, Iñigo Prieto, Daniela Zuluaga, Deliana Ropero, Neelesh Dewan, Jonathan D. Kirsch

**Affiliations:** 1 Epidemiology and Population Health Research Group (GESP), School of Public Health, Universidad del Valle, Cali, Colombia; 2 Department of Radiology, School of Medicine, Universidad del Valle, Cali, Colombia; 3 Division of General Internal Medicine, Department of Internal Medicine, University of Minnesota Medical School, Minneapolis, MN 55455 USA; 4 Department of Internal Medicine, University of Miami Miller School of Medicine, Miami, FL USA

**Keywords:** Dengue, Ultrasound, Plasma leakage, Primary care, Colombia

## Abstract

**Background:**

Early identification of plasma leakage may guide treatment decisions in dengue patients. This study evaluated the value of point-of-care ultrasound (POCUS) to detect plasma leakage and predict hospitalization or referral to a higher level of care in suspected dengue patients under routine conditions at a primary care facility in Colombia.

**Methods:**

We conducted a cohort study between April 2019 and March 2020 in a primary care hospital in Cali, Colombia. We prospectively included and followed 178 patients who were at least 2 years old with fever of less than 10 days and clinician-suspected dengue. A trained general practitioner performed a standardized POCUS protocol. Images were quality-rated and overread by an expert radiologist, and her results and those of the general practitioner were compared using the Kappa index. Logistic regression was used to identify factors associated with plasma leakage at enrollment and explore its prognostic value regarding hospital admission or referral to a higher level of care.

**Results:**

Half (49.6%) POCUS images were of suitable quality to be interpreted. The proportion of plasma leakage reported by the radiologist was 85.1% (95% *CI*: 78.6–90.2%) and 47.2% by the study physician (Kappa = 0.25, 95% *CI*: 0.15–0.35). The most frequent ultrasound findings were ascites (hepatorenal 87.2%, splenorenal 64%, or pelvic 21.8%) and gallbladder wall thickening (10.5%). Plasma leakage was higher in subjects with thrombocytopenia (a*OR* = 4, 95% *CI*: 1.3–12.1) and lower in patients 30–59 years old (a*OR* = 0.1, 95% *CI*: 0.0–0.4) than in those 18 years old or younger. POCUS evidence of plasma leakage (a*OR* = 8.2, 95% *CI*: 2.2–29.9), thrombocytopenia (a*OR* = 6.3, 95% *CI*: 2.4–16.0) and pulse pressure (a*OR* = 1.1, 95% *CI*: 1.07–1.2) were associated with hospital admission or referral to a higher level of care.

**Conclusions:**

Ultrasound is useful to detect plasma leakage in primary care and, challenges remain to guarantee high-quality images and diagnostic accuracy, for which a standardized dengue POCUS protocol and training program is needed.

**Graphical Abstract:**

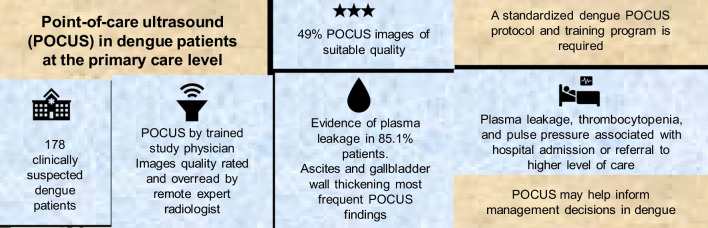

**Supplementary Information:**

The online version contains supplementary material available at 10.1186/s40249-023-01141-9.

## Background

Dengue is the most important arboviral disease worldwide in terms of morbidity, mortality, and economic impact [1]. In Colombia, dengue is a public health concern due to the infestation of the *Aedes aegypti* mosquito in more than 80% of the country, the introduction of *Aedes albopictus*, the cocirculation of the four serotypes, and the increased frequency of outbreaks, notably the epidemics of 1998, 2002, 2010, 2013 and 2019 [2]. In 2022, of the 69,947 dengue cases reported in Colombia, 46.8% were classified as dengue without warning signs, 51.2% dengue with warning signs, and 2.0% severe dengue [3].

In the natural history of dengue, plasma leakage generally occurs in patients in the critical or defervescence phase, but most patients seek medical attention for the first time in the preceding febrile phase. Early identification of subclinical signs of plasma leakage, together with clinical and laboratory criteria, could help guide therapeutic decisions and reduce the risk of complications, although the evidence is still unconclusive [4]. Plasma leakage can be detected by ultrasound and includes gallbladder wall thickening, pericholecystic fluid, perirenal, pararenal, hepatic and splenic subcapsular free fluid collection. Thoracic manifestations of plasma leakage in dengue can consist of pleural and pericardial effusions and pulmonary B-lines suggestive of pulmonary edema [5].

Standard ultrasound services are generally provided by radiology departments or other medical specialists (e.g., emergency medicine, internal medicine, obstetrics/gynecology) in secondary or tertiary levels of care. However, point-of-care ultrasound (POCUS) can be used by practitioners in the first levels of care given the advances in technology and inclusion of POCUS training in medical schools [6, 7]. Plasma leakage detection using POCUS by primary and emergency clinicians attending dengue patients could help improve treatment decisions in hyperendemic areas. However, there is insufficient evidence on the requirements for training general practitioners to perform POCUS in dengue patients, on the frequency of plasma leakage in dengue patients who seek care in primary care, and on the potential use of POCUS to inform treatment decisions in the first levels of care. Hence, the objectives of this study were (1) to determine the degree of agreement on ultrasound findings between a trained study physician and an expert radiologist, (2) to determine the frequency and factors associated with signs of plasma leakage using POCUS in patients with suspected dengue, and (3) to explore the value of POCUS in determining the risk of hospital admission or referral to a higher level of care. We conducted a pragmatic study under routine conditions at a primary health care facility in Colombia where a radiologist might be able to remotely read ultrasound images, a basic laboratory is available, including rapid dengue diagnostics, and most dengue cases are clinically diagnosed.

## Methods

### Study design

A pragmatic prospective cohort study was performed between April 2019 and March 2020 in a primary care public hospital in Cali, Colombia. The hospital’s clinical staff was asked to inform the research team (a study physician and a field assistant) when they encountered a patient with suspected dengue. Clinicians considered patients to have probable dengue (fever plus two nausea/vomiting, rash, aches/pains, tourniquet test positive, leukopenia, any warning sign), dengue with or without warning signs (abdominal pain/tenderness, persisting vomiting, clinical fluid accumulation, mucosal bleed, lethargy/restlessness, liver enlargement > 2 cm, increase hematocrit with decrease in platelet count), or severe dengue (severe plasma leakage leading to shock or respiratory distress, severe bleeding, and severe organ involvement) according to the 2009 World Health Organization (WHO) criteria [8]. No dengue confirmatory tests were performed as part of the research to reflect real-life conditions in the primary care setting.

We consecutively recruited men and women older than 2 years of age with reported fever of less than 10 days duration and diagnosis of dengue by their treating physician. Exclusion criteria included current pregnancy, contraindication to ultrasound (e.g., overlying skin injury in the region to be scanned), fever explained by another cause, comorbidities that result in third-spacing (e.g., liver failure, heart failure, cancer), and any condition requiring immediate attention that ultrasound examination would delay. Eligible participants underwent ultrasound examination and were followed up two weeks after enrollment to determine their clinical outcome. Sample size was estimated at 369 subjects based on a 40% proportion of plasma leakage at enrollment (20% less than the proportion reported in a tertiary care center) [9], a significance level of 5%, and precision of 5%. Recruitment ended in March 2020 secondary to the lockdown declared by the national government in response to the COVID-19 pandemic. To prevent our study from influencing clinical management, ultrasound findings were not shared with the treating physician unless it was to suggest considering the patient for further imaging studies. Ethical approval of this study was granted by the Ethics Committee of Universidad del Valle and the Hospital Joaquín Paz Borrero (HJPB) and the University of Minnesota (STUDY00004437). Written informed consent and, in the case of minors (< 18 years of age), written assent from the parent/guardian was obtained for all participants.

### Data collection

Data were collected using REDCap electronic data capture tools hosted at University of Minnesota [10] in a predesigned and encrypted case record form. First, the study general physician obtained demographic information and performed a clinical interview and physical examination to determine the participants’ dengue classification [8]. Then, POCUS was performed following a standardized protocol based on the Focused Assessment with Sonography for Trauma (FAST) exam [11]. The results of laboratory work-up throughout an admission were recorded into REDCap. The laboratory results requested by the treating physician, such as rapid dengue IgM and IgG tests (SD BIOLINE Dengue IgG/IgM, Standard Diagnostics, Republic of Korea) together with maximum hematocrit and monocytes and minimum leukocyte, lymphocyte, and platelet values for the current episode, were recorded. NS1-based tests were not available at the health facility. Participants’ clinical course, need for hospital admission or referral to a higher level of care, and final diagnosis were gathered via telephone follow-up approximately 14 days after study enrollment. Outcomes of those unreachable by telephone were ascertained via review of medical records.

### Ultrasound examination

#### Training

The study general physician, who did not have any prior training in ultrasound, underwent focused training in performance and interpretation of POCUS in accordance with guidelines published by the Society of Point of Care Ultrasound and American College of Emergency Physicians (ACEP) prior to study commencement [12, 13]. This included a 3-hour online module on general ultrasound principles, correct technique, and image interpretation followed by 30 h (over 5 days) of practice sessions on 4 healthy volunteers and 53 hospitalized patients with mainly cardiovascular diseases, including cardiac failure but not dengue. The trainee obtained a total of 627 POCUS images. Training was conducted in Cali, Colombia, by a single provider, a U.S.-based emergency medicine physician and a trainer of ultrasonography at Hennepin County Medical Center and the University of Minnesota. An additional 6-hour practice session was administered by the study expert radiologist 10 months after study initiation with the aim of improving technique and image interpretation.

#### Equipment, image recording and interpretation

A Philips Lumify C5-2 broadband curved array transducer connected to a Samsung Galaxy Tab S5 operating the Philips Lumify Ultrasound app was utilized for training and study data collection. Three-second video recordings and still images of ultrasound scans were saved locally on the tablet’s password-secured hard drive and later uploaded to Box for Healthcare, a cloud-based storage platform compliant with the US Health Insurance Portability and Accountability Act of 1996. Ultrasound video recordings and stills were independently interpreted by both the trained general physician and study expert radiologist. Image quality was assessed by the radiologist using the ACEP grading system [14] (see Additional file 1 for detailed definition of ACEP grading system).

#### Standardized protocol

**Fig. 1:**
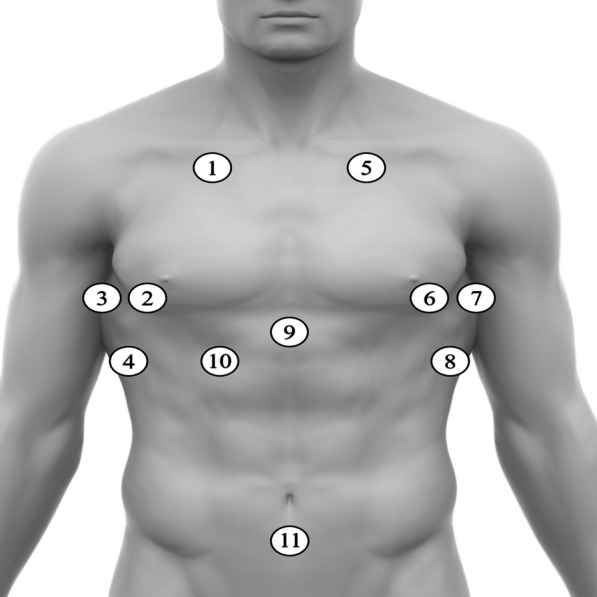
Sites examined by point-of-care ultrasound for evidence of plasma leakage in patients with suspected dengue

The standardized POCUS protocol utilized in this study was developed according to preliminary data on ultrasound findings in dengue patients [5]. First, the patient is placed in a semi recumbent position at 45° or supine (if the participant does not tolerate 45°) regardless of fasting time. The protocol first assesses the presence of pulmonary B-lines in the lungs’ apexes at the intersection of the midclavicular line and second intercostal space and in the lungs’ bases at the intersection of the midclavicular line and fourth to fifth intercostal space. Assessment of pleural effusions occurs at the intersection of the posterior axillary line and sixth to ninth intercostal space, whichever best captures the costophrenic angle. Pericardial effusion is assessed via a subxiphoid approach and with a transverse probe orientation. Free fluid in the hepatorenal space is assessed at the mid- to posterior axillary line typically at the eighth to eleventh rib spaces, in the splenorenal space at the mid- to posterior axillary line typically at the sixth to ninth rib spaces, and in the pelvic space at the midline superior to the pubic symphysis and with a sagittal probe orientation. The appearance and thickness of the anterior wall gallbladder was assessed at its variable location in the right upper quadrant using in-app calipers. The order in which the protocol was implemented went from the right to the left side and then to the center part of the body to maximize sonographer and patient comfort and minimize exam duration (Fig. 1).

### Statistical analysis

Absolute and relative frequencies were estimated for categorical variables and means with corresponding standard deviation or median and range for quantitative variables according to their distribution. Thrombocytopenia was defined as platelet counts < 100,000 per mm^3^, leukopenia as < 4000 leukocytes/µl, lymphopenia as < 1000 lymphocytes/µl and monocytosis as > 1200 monocytes/µl. The degree of hemoconcentration was calculated by subtracting the minimum hematocrit from the peak hematocrit recorded, then dividing the result by the minimum hematocrit and multiplying by 100. We used in the analysis the dengue classification made by the study physician. All analyses were performed considering only those images with a quality score of 3 (“Minimal criteria met for diagnosis, recognizable structures but with some technical or other flaws”) or more. The Kappa coefficient was used to determine the degree of interobserver agreement between the study physician and the expert of plasma leakage overall and by anatomical site. The Kappa coefficient was interpreted as follows: 0.01–0.20 = “slight”, 0.21–0.40 = “fair”, 0.41–0.60 = “moderate”, 0.61–0.80 = “substantial” and 0.81–0.99 = “almost perfect” [15].

The frequency of plasma leakage was determined as the number of participants with ultrasonographic evidence of pleural effusion (any volume), B-lines (> 3 in either apex or base of both lungs), pericardial effusion, ascites, pericholecystic fluid, and thickened gallbladder wall (> 3 mm) reported by the expert radiologist divided by the total number of participants examined with at least one image of suitable quality (score 3 or more). The associations between evidence of plasma leakage as interpreted by the expert radiologist and sociodemographic, clinical, and laboratory characteristics were determined by calculating crude and adjusted *OR*s (c*OR*s and a*OR*s) with 95% confidence intervals (*CI*s). The same approach was used for factors predicting hospital admission or referral in comparison to outpatient care. Categorical variables were compared using the chi-squared or Fisher exact test as appropriate, and quantitative variables were compared using Student's *t* test or nonparametric tests. Multiple logistic regression models were fitted using backward selection, considering the statistical and clinical relevance of variables, and the likelihood-ratio test. The goodness-of-fit of the regression models was assessed with the Hosmer and Lemeshow test, and predicted probabilities were estimated using average marginal effects. A *P* value < 0.05 was considered statistically significant. Analyses were performed in STATA 14 (Stata-Corp, College Station, TX, USA).

## Results

### Characteristics of patients

**Fig. 2:**
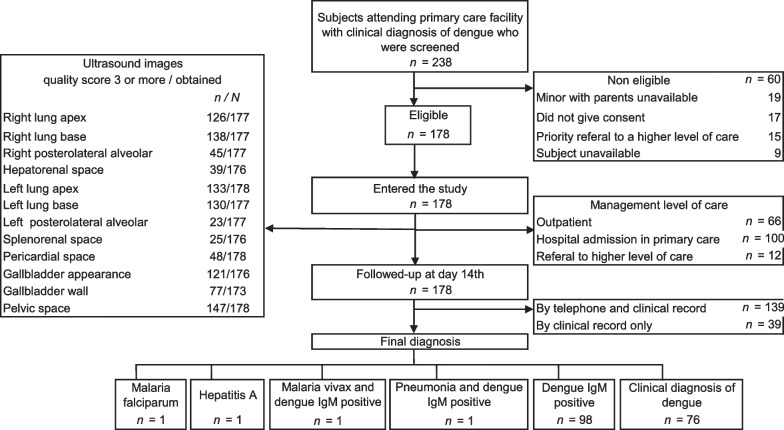
Flowchart of participant selection and follow-up

A total of 238 patients with clinical diagnoses of dengue were screened, and 178 entered the study. Follow-up was completed by telephone in 139 (78%) participants and in 39 (22%) only by reviewing clinical records (Fig. 2). There were 44.4% men, with a median age of 16.2 (range 2–85) years, a median of 5 (0–10) days of fever onset, and none with pulse pressure < 20 mmHg at enrollment. While none of the participants were clinically classified as severe dengue, more than half (*n* = 98, 55%) were clinically classified as dengue with warning signs. Those with warning signs were more often female (61.2%), 30 years old or younger (87.8%), with fever onset of one week or more (40.8%), and with thrombocytopenia (78.5%). A dengue IgM rapid test was performed in almost all (155/178, 87%) participants, with positive results in 64.5% (100/155) of them. A dengue IgG rapid test was reported less frequently than IgM (54% of participants), with positive results in 67.7% (65/96). The most frequent hemogram anomaly was thrombocytopenia (73.6%), followed by leukopenia (51.7%), lymphopenia (16.8%), and monocytosis (13.4%). The highest degree of hemoconcentration was 59.2% in an IgM-positive subject aged 32 years old, with a minimum hematocrit of 18.2% and a maximum of 29.0% (Table 1). Other clinical tests were seldom requested, including the following: aspartate aminotransferase/alanine aminotransferase (*n* = 45), blood creatinine (*n* = 17), blood urea nitrogen (*n* = 9), malaria thick smear (*n* = 4), blood sodium (*n* = 2), and chest X-ray (*n* = 1).

**Table 1:** Characteristics of the study population and point-of-care ultrasound findings according to dengue clinical classification

Characteristic	Total	Dengue without warning signs	Dengue with warning signs	
	*n* = 178 (%)	*n* = 80 (%)	*n* = 98 (%)	*P*-value
Males	79 (44.4)	41 (51.2)	38 (38.8)	0.09
Age in years				
Median (range)	16.5 (2–85)	17 (2–85)	16 (2–60)	0.11
2–18	103 (57.9)	43 (53.7)	60 (61.2)	0.002
19–29	37 (20.8)	11 (13.7)	26 (26.6)	
30–59	28 (15.7)	17 (21.2)	11 (11.2)	
60 or more	10 (5.6)	9 (11.2)	1 (1)	
Days of fever onset				
Median (range)	5 (0–10)	5 (0–10)	5 (1 -10)	0.23
0–3	24 (13.4)	15 (18.8)	9 (9.2)	0.11
4–6	90 (50.6)	41 (51.2)	49 (50)	
7–10	64 (36)	24 (30)	40 (40.8)	
Mean arterial pressure mmHg^a^	80 (61.6–121.6)	80 (61.6–103.3)	80 (65.0–121.6)	0.31
Pulse pressure mmHg^a^	35 (20–80)	40 (20–80)	35 (25–70)	0.003
Dengue IgM	*n* = 155	*n* = 67	*n* = 88	
Negative	55 (35.5)	26 (38.8)	29 (33)	0.45
Positive	100 (64.5)	41 (61.2)	59 (67)	
Dengue IgG	*n* = 96	*n* = 43	*n* = 53	
Negative	31 (32.3)	15 (34.9)	16 (30.2)	0.62
Positive	65 (67.7)	28 (65.1)	37 (69.8)	
Dengue IgM/IgG	*n* = 96	*n* = 43	*n* = 53	
Both negative	19 (19.8)	9 (21)	10 (18.8)	0.96
Both positive	48 (50)	21 (48.8)	27 (51)	
Positive/negative	12 (12.5)	6 (14)	6 (11.3)	
Negative/positive	17 (17.7)	7 (16.2)	10 (18.9)	
Maximum hematocrit (%) ^a^	42.7 (27.3–54.7)	42.5 (27.3–54.7)	42.8 (27.8–54.6)	0.93
	*n* = 170	*n* = 74		
Hemoconcentration (%) ^a^	9.6 (0–59.2)	9.9 (0–30.3)	9.4 (0–59.2)	0.76
Leukocytes (cells × 10^3^/µl)^a^	3.9 (1–49)	3.9 (1–14.1)	3.7 (1.4–49.0)	0.51
Leukopenia (< 4000 cells × 10^3^/µl)	92 (51.7)	40 (50)	52 (53%)	0.68
Lymphocytes (cells × 10^3^/µl)^a^	1.73 (0.39–9.09)	1.65 (0.43–9.09)	1.85 (0.39–3.92)	0.56
Lymphopenia (< 1000 cells × 10^3^/µl)	30 (16.8)	15 (18.7)	15 (15.3)	0.54
	*n* = 176		*n* = 96	
Monocytes (cells × 10^3^/µl)^a^	0.49 (0–2.4)	0.5 (0–2.0)	0.47 (0–2.4)	0.74
Monocytosis (> 1200 cell × 10^3^/µl)	24 (13.4)	11 (13.7)	13 (13.2)	0.92
Platelets (cells/mm^3^)^a^	73,000 (12,000–283,000)	70,000 (19,000–268,000)	73,000 (12,000–283,000)	0.72
Thrombocytopenia (< 100,000 cells/mm^3^)	131 (73.6)	54 (67.5)	77 (78.5)	0.09
POCUS findings	*n* = 161	*n* = 73	*n* = 88	
Any evidence of plasma leakage	137 (85.1)	61 (83.6)	76 (86.4)	0.64
Number of anatomical sites positive^a^	2 (0–7)	1 (0–7)	2 (0–7)	0.08
Hepatorenal ascites	34/39 (87.2)	13/14 (92.9)	21/25 (84)	0.42
Splenorenal ascites	16/25 (64)	3/8 (37.5)	13/17 (76.4)	0.05
Pelvic ascites	32/147 (21.8)	10/66 (15.1)	22/81 (27.1)	0.08
Gallbladder wall thickening > 3 mm	8/76 (10.5)	2/30 (6.6)	6/46 (13)	0.37
Pericholecystic fluid	7/121 (5.8)	0/51	7/70 (10)	0.02
Right sided pleural effusion	4/45 (9)	3/24 (12.5)	1/21 (4.7)	0.36
Pericardial effusion	3/48 (6.2)	2/24 (8.3)	1/24 (4.1)	0.55
Left sided pleural effusion	1/23 (4.3)	1/9 (11.1)	0/14	0.20
B lines > 3	3/96 (3)	2/40 (5)	1/56 (1.8)	0.37

^a^Median (range)

Denominators indicate the number of POCUS images of suitable quality for the corresponding anatomical site

### Ultrasound image quality

**Fig. 3:**
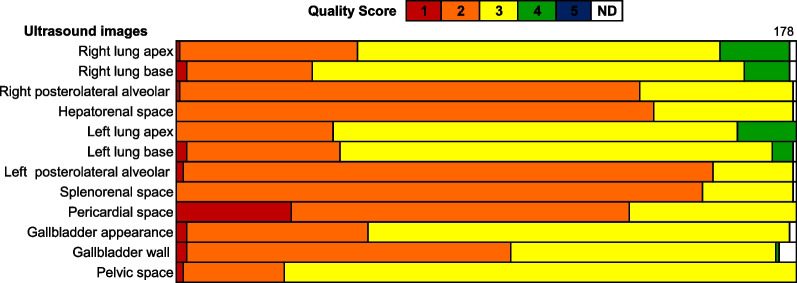
Quality score of point-of-care ultrasound images by anatomical site. *ND* not done

A total of 2121 images were obtained by the study physician and quality scored by the radiologist. Of these, 1053 (49.6%) corresponding to 161 patients were of suitable quality (score 3 or more) to be interpreted for evidence of plasma leakage by the expert radiologist (Fig. 3). Overall quality was independent of the patient’s age, sex, and dengue classification. Except in the right lung apex where the quality was higher for younger patients, in the right lung base where quality was higher for men, and the gallbladder wall where quality was higher for women (see Additional File 2 for results of image quality by patient characteristics). The median fasting time before POCUS was 3 h (IQR: 1–5).

### Ultrasound results

**Fig. 4:**
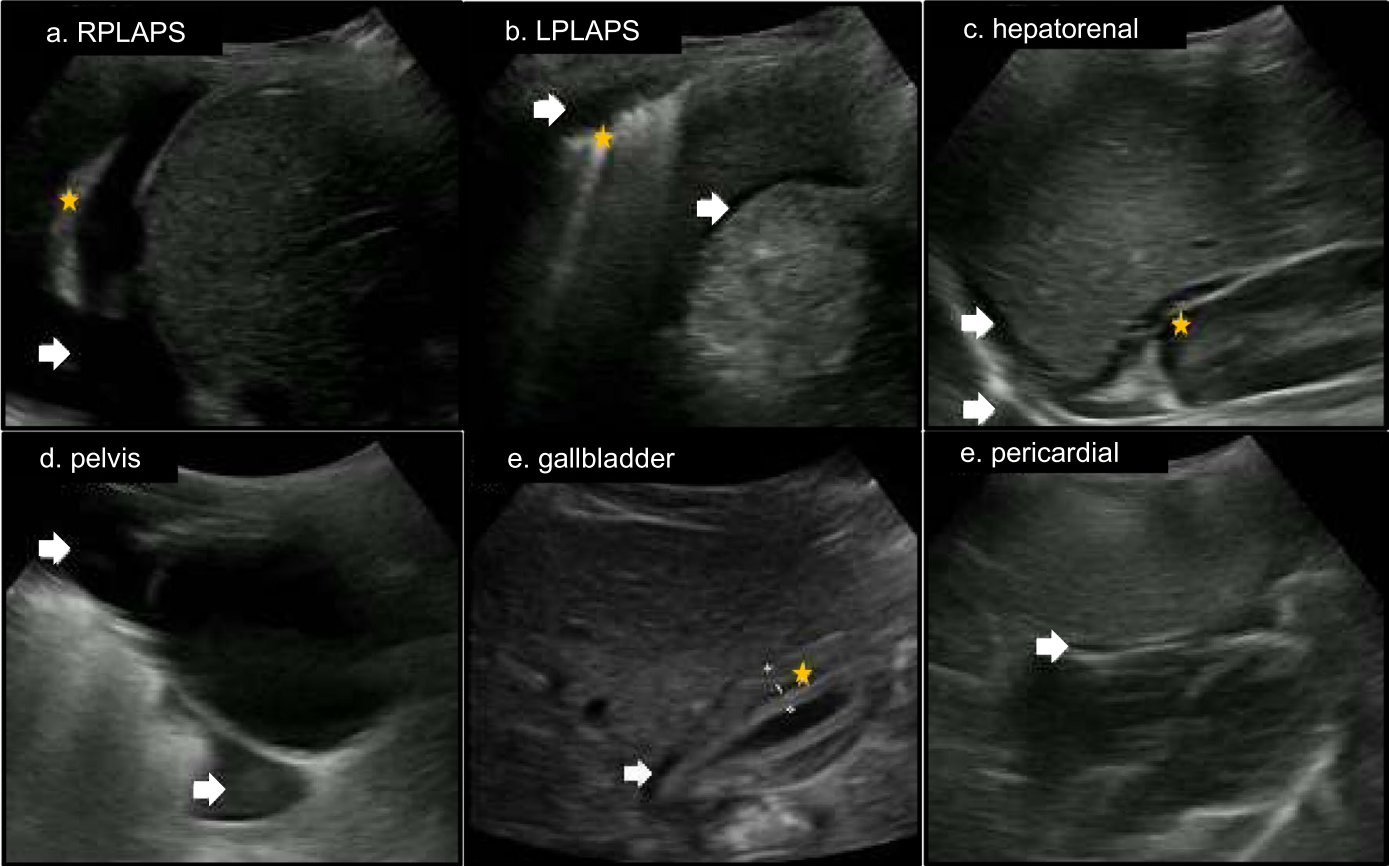
Point-of-care ultrasound findings suggesting plasma leakage. **a** RPLAPS right posterolateral alveolar or pleural syndromes-point demonstrating pleural effusion (arrow) and lung atelectasis (star); **b** LPLAPS left posterolateral alveolar or pleural syndromes-point demonstrating lung atelectasis (star), pleural effusion (left arrow), and splenorenal ascites (right arrow); **c** hepatorenal ascites (top arrow), right pleural effusion (bottom arrow), and perirenal fluid (star); **d** pelvis demonstrating abdominal ascites (top arrow) and liquid in rectovesical pouch (bottom arrow); **e** gallbladder with calipers measuring gallbladder wall (star) thickness and demonstration of pericholecystic fluid (arrow); **f** pericardial effusion (arrow)

Plasma leakage was observed in 85.1% (95% *CI*: 78.6–90.2%) of participants and was more frequent in the abdomen, manifesting as ascites (hepatorenal or splenorenal or pelvic) and/or gallbladder wall thickening (Table 1) (Fig. 4). The appearance of the gallbladder was reported as normal in 84/121 (69.4%) subjects, followed by striated and thickened in 11 (9.1%), contracted in 10 (8.2%), with a “honeycomb” pattern and thickened in 7 (5.8%), with pericholecystic fluid and striated in 4 (3.3%), “honeycomb” and pericholecystic fluid in 3 (2.5%) and thickened in 2 (1.7%). Splenorenal ascites, pelvic ascites, gallbladder wall thickening, and pericholecystic fluid were more frequent in subjects classified as dengue with warning signs, but only the latter was statistically significant (*P* = 0.02) (Table 1).

### Ultrasound agreement

The study physician detected plasma leakage in 76 (47.2%) subjects, which corresponds to a Kappa value of 0.25 (95% *CI*: 0.15–0.35) considered “fair”. The disagreement was particularly observed in the ascites, where the study physician detected a smaller proportion of the splenorenal (4/25 vs 16/25, Kappa = 0.06) and hepatorenal (5/39 vs 34/39, Kappa = 0.04) ascites than the radiologist. There was improved concordance for right pleural effusion (5/45 vs 4/45, Kappa = 0.18), pericardial effusion (3/48 vs 3/48, Kappa = 0.28), pelvic ascites (23/146 vs 32/146, Kappa = 0.48) and even better for the B-lines, left pleural effusion and thickened gallbladder wall, where the study physician and radiologist detected a similar proportion (2/96 vs 3/96, Kappa = 0.79), (2/23 vs 1/23, Kappa = 0.64), and (7/77 vs 8/77, Kappa = 0.62), respectively.

### Factors associated with plasma leakage

A decreased frequency of plasma leakage was observed in the age group 30 to 59 when compared to those ≤ 18 years old. Patients ≤ 18 years old showed more pelvic ascites (27/87, 31%) than older patients (5/60, 8.3%) (*P* = 0.001). In contrast, patients > 18 years old were more likely to show B-lines (4/68, 5.8%) than younger patients (0/94) (*P* = 0.01) There was no association between POCUS evidence of plasma leakage and sex, dengue clinical classification, leukopenia, lymphopenia, or monocytosis. Plasma leakage was more frequent in those seeking care with 7 to 10 days of fever onset who had hemoconcentration and thrombocytopenia. Of these, thrombocytopenia and age had a statistically significant association in the multivariate model (Table 2). The final multivariate model demonstrated a good fit (*P* = 0.16) with McFadden’s R^2^ of 0.20.

**Table 2:** Factors associated with point-of-care ultrasound evidence of plasma leakage in suspected dengue patients

Characteristic	Plasma leakage in POCUS		c*OR* (95% *CI*)	*P*-value	a*OR* (95% *CI*)	*P-*value
	Yes *n* = 137(%)	No *n* = 24 (%)				
Female	76 (55.5)	12 (50)	1.2 (0.5–3)	0.62		
Age in years						
2–18	83 (60.6)	11 (45.8)	1		1	
19–29	31 (22.6)	3 (12.5)	1.4 (0.3–5.4)	0.64	1.1 (0.2–4.6)	0.92
30–59	15 (11)	9 (37.5)	0.2 (0.07–0.6)	0.004	0.1 (0.0–0.5)	0.001
60 or more	8 (5.8)	1 (4.2)	1.06 (0.1–9.3)	0.95	0.7 (0.1–7.2)	0.78
Dengue without warning signs	61 (44.5)	12 (50)	1			
Dengue with warning signs	76 (55.5)	12 (50)	1.2 (0.5–3)	0.62		
Days of fever onset						
0–3	15 (11)	6 (25)	1			
4–6	68 (49.6)	12 (50)	2.3 (0.7–7,1)	0.15		
7–10	54 (39.4)	6 (25)	3.6 (1–12.8)	0.04		
Dengue IgM/IgG	*n* = 74	*n* = 10				
Both negative	12 (16.4)	3 (30)	1			
Both positive	39 (52.4)	2 (20)	4.8 (0.7–32.6)	0.10		
Positive/negative	8 (11)	2 (20)	1 (0.1–7.3)	1.00		
Negative/positive	14 (19.2)	3 (30)	1.16 (0.2–6.9)	0.17		
		*n* = 23				
Hematocrit maximum (%) Me (range)	42.5 (27.3–54.7)	44.3 (33.6–52.7)	0.9 (0.8–1.0)	0.09		
	*n* = 134	*n* = 20				
Hemoconcentration (%) Me (range)	10.1 (0–59.2)	6.6 (0–20.4)	1.1 (1.0–1.2)	0.01	1.1 (0.9–1.2)	0.07
Leukopenia (< 4000 cells × 10^3^/µl)	70 (51.1)	12 (50)	1.04 (0.4–2.5)	0.92		
Lymphopenia (< 1000 cells × 10^3^/µl)	22 (16.1)	7 (29.1)	0.46 (0.2–1.2)	0.13		
Monocytosis (> 1200 cell × 10^3^/µl)	21 (15.3)	1 (4.1)	4.1 (0.5–32.5)	0.17		
Thrombocytopenia (< 100,000 cells/mm^3^)	110 (80.3)	10 (41.6)	5.7 (2.2–14.2)	< 0.001	4.1 (1.3–12.4)	0.01

Final multiple model includes age, hemoconcentration and thrombocytopenia

### Factors associated with hospital admission or referral

In two subjects, the treating physician concluded that the diagnosis was not dengue; one was diagnosed with malaria due to *Plasmodium falciparum* and had no POCUS evidence of plasma leakage, while the other was diagnosed with hepatitis A and had evidence of hepatorenal ascites. Two other study participants were diagnosed as having coincident dengue and another disease; one was diagnosed with malaria due to *P. vivax* with the presence of pelvic ascites, and another with pneumonia with no signs of plasma leakage. All participants were alive at day 14 after enrollment, 66 (37.1%) were treated as outpatients, 100 (56.2%) were hospitalized, and 12 (7.7%) required referral to a higher level of care due to the severity of their condition. POCUS evidence of plasma leakage was associated with hospital admission or referral (c*OR* = 7.5, 95% *CI*: 2.7–20.4, *P* < 0.0001), as was thrombocytopenia (6.1, 95% *CI*: 3–12.6, *P* < 0.0001), a unit increase in pulse pressure (1.04, 95% *CI*: 1–1.07, *P* = 0.02) and degree of hemoconcentration (1.07, 95% *CI*: 1.01–1.13, *P* = 0.01) (Table 3). All these variables, except hemoconcentration, were associated with hospital admission or referral to a higher level of care in the multivariate model, while older age was associated with outpatient care (Table 3). The final model demonstrated a good fit (*P* = 0.7) with McFadden’s R^2^ of 0.27. The predicted probability of being hospitalized if there was POCUS evidence of plasma leakage on admission was 0.70 (95% *CI*: 0.63–0.76) and 0.32 (95% *CI*: 0.13–0.51) without POCUS evidence of plasma leakage. The total number of POCUS anatomical sites positive for plasma leakage was associated with hospital admission or referral to a higher level of care (Table 3) but did not enter the final model because it was correlated with POCUS evidence of plasma leakage. A single positive POCUS result (regardless of the anatomical site) showed 93.3% sensitivity and 33.3% specificity for hospitalization/referral. With each additional positive site, sensitivity decreased, and specificity increased. Of the POCUS findings, hepatorenal ascites (c*OR* = 3.8, 95% *CI*: 0.5–29.3, *P* = 0.2) showed an increased risk of hospital admission or referral to a higher level of care, but it did not reach statistical significance (Table 3).

**Table 3:** Factors associated with hospital admission or referral to a higher level of care in suspected dengue patients

Characteristic	Hospitalized		c*OR* (95% *CI*)	*P-*value	a*OR* (95% *CI*)	*P-*value
	Yes *n* = 112 (%)	No *n* = 66 (%)				
Female	65 (58)	34 (51.5)	1.3 (0.7–2.4)	0.39		
Age in years						
2–18	67 (59.8)	36 (54.5)	1		1	
19–29	24 (21.4)	13 (19.7)	1 (0.4–2.1)	0.98	0.3 (0.1–0.9)	0.04
30–59	16 (14.3)	12 (18.2)	0.7 (0.3–1.6)	0.44	0.4 (0.1–1.5)	0.19
60 or more	5 (4.5)	5 (7.6)	0.5 (0.1–1.9)	0.35	0.02 (0.002–0.2)	0.001
Dengue without warning signs	50 (44.6)	30 (45.5)	1			
Dengue with warning signs	62 (55.4)	36 (54.5)	1.0 (0.5–1.9)	0.91		
Days of fever onset						
0–3	13 (11.6)	11 (16.6)	1			
4–6	62 (55.4)	28 (42.4)	1.8 (0.7–4.7)	0.18		
7–10	37 (33)	27 (41)	1.1 (0.4–3.0)	0.76		
Pulse pressure mmHg	40 (25–80)^a^	35 (20–55)^a^	1.0 (1.0–1.1)	0.02	1.1 (1.07–1.2)	< 0.0001
Any evidence of plasma leakage by POCUS	98/104 (94.2)	39/57 (68.4)	7.5 (2.7–20.4)	< 0.0001	8.2 (2.2–29.9)	0.001
Number of POCUS anatomical sites positive for plasma leakage	2 (0–7)	1 (0–6)	1.5 (1.1–2.0)	0.002		
Hepatorenal ascites	29/32 (90.6)	5/7 (71.4)	3.8 (0.5–29.3)	0.2		
Splenorenal ascites	10/18 (55.5)	6/7 (85.7)	0.2 (0–2.1)	0.1		
Pelvic ascites	24/98 (24.5)	8/49 (16.3)	1.6 (0.6–4.0)	0.2		
Gallbladder wall thickening > 3 mm	5/53 (9.4)	3/23 (13)	0.7 (0.1–3.1)	0.6		
Pericholecystic fluid	5/78 (6.4)	2/43 (4.6)	1.4 (0.2–7.5)	0.7		
Gallbladder with "honeycomb" pattern	7/78 (9.0)	3/43 (7.0)	1.3 (0.3–5.3)	0.7		
Right sided pleural effusion	2/27 (7.4)	2/18 (11.1)	0.6 (0.1–5.0)	0.6		
Pericardial effusion	2/30 (6.7)	1/18 (5.5)	1.2 (0.1–14.4)	0.8		
Left sided pleural effusion	1/16 (6.2)	0/7	–			
B lines > 3	2/69 (3.0)	1/27 (3.7)	0.5 (0.1–3.9)	0.5		
Dengue IgM/IgG						
Both negative	10/56 (17.9)	9/40 (22.5)	1			
Both positive	32/56 (57.1)	16/40 (40)	1.8 (0.6–5.3)	0.28		
Positive/negative	4/56 (7.1)	8/40 (20)	0.4 (0.1–2.0)	0.29		
Negative/positive	10/56 (17.9)	7/40 (17.5)	1.2 (0.3–4.8)	0.70		
Maximum hematocrit (%) Me (range)	42.7 (27.8–54.7)	42.7 (27.3–52.7)	1.0 (0.9–1.1)	0.58		
Hemoconcentration (%) Me (range)	10.8 (0–59.2)	8.3 (0–24.1)	1.0 (1.0–1.1)	0.01		
Leukopenia (< 4000 cells × 10^3^/µl)	57 (51)	35 (53)	0.9 (0.5–1.6)	0.78		
Lymphopenia (< 1000 cells × 10^3^/µl)	21 (18.7)	9 (13.6)	1.4 (0.6–3.4)	0.38		
Monocytosis (> 1200 cell × 10^3^/µl)	17 (15.2)	7 (10.6)	1.5 (0.6–3.8)	0.39		
Thrombocytopenia (< 100,000 cells/mm^3^)	97 (86.6)	34 (51.5)	6.1 (3.0–12.6)	< 0.001	6.3 (2.4–16.0)	< 0.0001

^a^Median (range)

Final multiple model includes age group, unit increase in pulse pressure, plasma leakage by POCUS, and thrombocytopenia

## Discussion

In this study, we sought to detect plasma leakage using remote radiologist-interpreted POCUS in clinically diagnosed dengue patients in a primary care facility in an endemic area in Colombia. Despite the limited sample size, POCUS evidence of plasma leakage at the time of consultation was associated with hospital admission or referral during follow-up. Hospital admission as a proxy of severe dengue has several limitations [16], but whether to admit patients or refer them to higher levels of care is an important practical management decision in primary care [17]. To date, there is insufficient evidence on the predictive value of ultrasound in severe dengue [4]. A recent cohort study found that an abnormal ultrasound on day 1 of admission was associated with the development of clinical characteristics of severe dengue. They also reported that all severe dengue patients had thrombocytopenia (< 150 × 10^9^/L) on admission, but the study included only adults and did not assess other predictive factors [18]. Another cohort in a pediatric population with suspected dengue found both gallbladder wall thickening (> 3 mm) and positive abdominal fluid associated with hospital admission at presentation. Gallbladder wall thickening on presentation was also associated with a return visit or hospitalization during follow-up [19]. In addition, pleural effusion and cardiovascular instability have been associated with admission to the intensive care unit of pediatric dengue patients [20]. In the primary care setting, physicians are trained to follow national guidelines based on those of the WHO to admit patients with warning signs, signs of plasma leakage (e.g., hypotension), evidence of spontaneous bleeding, organ involvement and other concomitant conditions such as pregnancy or another infection. These findings and ours support further research aimed at studying the potential use of both ultrasound evidence of plasma leakage and thrombocytopenia as predictors of severe dengue or further support the need for hospital admission/referral.

Most subjects (85.1%) had at least one ultrasound finding of plasma leakage, which falls between the 73.6% and 100% proportion reported in studies using ultrasound [21–24] . The frequency of ultrasound findings suggesting plasma leakage in our study contrasts with those of Vedaraju et al., who reported higher frequencies of gallbladder wall thickening (83.3% vs 10.5%) and right pleural effusion (20.6% vs 9%) but lower frequencies of ascites (53.9% vs 87.2%) and pericardial effusion (1.9% vs 6.2%) [24]. The varying frequency of plasma leakage on admission could be explained by the stage of the disease in the participants, the anatomical areas explored, and the ultrasound reader’s expertise. Findings in the gallbladder wall as either thickening (> 3 mm) or abnormal appearance (e.g., striated, thickened, contracted, and “honeycomb” pattern) are not specific since they can be seen in several diseases, such as cholecystitis, congestive heart failure, renal failure, liver disease, and pancreatitis [25]. However, when restricted to dengue patients, they have been found to be potentially useful to diagnose or predict dengue with warning signs or severe dengue [26–29]. This was confirmed in our study since abnormal appearance of the gallbladder wall (pericholecystic liquid) was associated with dengue with warning signs. Proper examination of the gallbladder wall requires appropriate fasting time, especially in adults [30]. This is difficult to guarantee in real life when implementing abdominal ultrasound in dengue patients in the emergency department and results in false positives. It has been suggested that increasing the cutoff to define gallbladder wall thickening from 3 mm to 4- or 5-mm increases specificity without decreasing sensitivity to detect or predict severe dengue in children, but this needs further validation [31]. Checking for ascites is part of the physical examination in a dengue patient, but it only detects a relatively large volume (> 500 ml) and can be difficult in obese patients. Ultrasound is considered an accurate complement to the physical examination of the abdomen in the emergency setting, but there is no protocol to use it in dengue patients as it is in trauma [11]. This has resulted in most dengue studies reporting unspecified ascites [5]. Pleural effusion can also be detected by physical examination or chest X-rays. In our study, it was a relatively rare ultrasound finding (with a frequency of less than 10% on the right or left side) and was not associated with the dengue clinical classification. These findings contrast with others where pleural effusion was the most frequent ultrasound finding when assessed daily and particularly in dengue hemorrhagic fever [32]. Pericardial effusion has been reported less frequently in adults (7.4%) [33] than in children (17.7%), in whom it is associated with severe dengue [34]. In any case, the results of implementing ultrasound in the routine care of dengue patients are likely to be influenced by the available technical expertise, timing in relation to the natural history of the disease, and frequency of ultrasound.

Regarding technical expertise, we found that the interobserver agreement was higher in the lungs than in the abdomen, pointing to the need to develop a reproducible specific dengue protocol and maintain proper standards of training for POCUS [12]. In 2022, consensus recommendations for ultrasound education of undergraduate medical students underlined that some images are more difficult to obtain than others because of difficult views in the anatomical sites and patient characteristics. Consequently, it is suggested that training starts with easier views to facilitate progress to more advanced skills to capture quality images [35]. In addition, the relatively high proportion of low-quality images obtained by the trained study physician points to the need to incorporate validated tools to assess competency in image quality during and at the end of POCUS training for dengue, as has been done for FAST [36].

Consistent with the clinical course of dengue and hyperendemic transmission in Colombia, days of fever, secondary dengue infection (defined as concurrent IgM and IgG positivity), young age, and thrombocytopenia were associated with plasma leakage. The first two factors did not enter the final adjusted model, likely due to insufficient statistical power. Plasma leakage was observed more frequently after day 4 of fever (when the critical phase is expected to start) and beyond day 7 (when the critical phase is expected to end) [37], perhaps because of inaccurate reporting of illness duration and the capacity of POCUS to detect plasma leakage after illness resolution. Secondary infection is a well-known risk factor for severe dengue mediated by antibody-dependent enhancement and influenced by the time interval between infections and dengue virus serotype [8]. Children have been found to have more plasma leakage as a sign of severe dengue than adults [37], and moderate to severe thrombocytopenia is a common laboratory finding during the critical phase [38]. Hemoconcentration was also associated with plasma leakage but did not enter the multivariate model, probably because it was correlated with thrombocytopenia. Some proposed dengue severity scores have shown the potential use of both the degree of thrombocytopenia and hemoconcentration, together with other laboratory tests, as markers of plasma leakage to help clinical decisions [39]. Implementing a simplified standard protocol of POCUS and hemogram in the primary care of dengue patients would be useful to validate such scores, as has been done in higher levels of care [40].

There are several limitations in our study. First, subjects were included based on clinical rather than confirmatory laboratory diagnosis of dengue, which causes misclassification of other diseases as dengue and vice versa. Clinical diagnosis of dengue tends to have more sensitivity than specificity, resulting in more false positives (i.e., other diseases diagnosed as dengue) than negatives (i.e., dengue diagnosed as other disease) [41]. Hence, the estimated proportion of plasma leakage would be influenced by whether the other diseases (misclassified as dengue) present with plasma leakage. Examples of these are the hepatitis A and malaria cases enrolled with and without POCUS evidence of plasma leakage, respectively. Despite this limitation, the fact that the study was conducted in a single health facility, with experienced clinicians in a dengue endemic area, and basic lab tests were available to help differential diagnosis and follow-up allow the identification of other diagnoses, overcoming the logistical restrictions of performing virological dengue diagnosis in primary care. Second, many POCUS images obtained by the trained general physician were not available for interpretation by the expert radiologist due to their low quality. It is not possible to anticipate the effect of not including these images in the study results. Third, we were unable to secure another POCUS expert to assess the reproducibility of the image quality rating and final interpretation performed by the study expert. In this setting, radiologists are not usually available. Hence, our pragmatic study reflects a real-life scenario in which either a trained general physician would acquire and interpret POCUS images, or a radiologist would remotely interpret the images acquired by the trained general physician. To control for this bias, the quality of the POCUS images was assessed, and only those considered suitable were interpreted by the radiologist who was masked to the subject’s clinical diagnosis and the general physician’s interpretation.

## Conclusions

Using remote radiologist-interpreted POCUS, plasma leakage in suspected dengue patients in primary care was frequent and associated with young age and thrombocytopenia (< 100,000 cells/mm^3^). Ascites (hepatorenal, splenorenal, and pelvic) and changes in the gallbladder (wall thickening > 3 mm and pericholecystic fluid) were the most frequent POCUS findings. POCUS evidence of plasma leakage at the time of patient presentation is a potential indicator of management decisions such as hospital admission and referral to higher levels of care. Further studies comparing single versus serial ultrasounds and validating clinical scores that include POCUS in dengue patients in primary care are warranted. Studies require a reproducible specific dengue POCUS protocol and proper standards of training and assessment of competence for primary care physicians.

### Supplementary Information

**Additional file 1: Table**. American College of Emergency Physicians (ACEP) suggested ultrasound image quality rating.

**Additional file 2: Table**. Results of point-of-care ultrasound image quality by age, sex, and dengue clinical classification of patients.

## Data Availability

The datasets generated and analyzed during the current study are available in Osorio, Lyda, 2023, "Replication Data for: Evaluation of remote radiologist-interpreted point-of-care ultrasound for suspected dengue patients in a primary health care facility in Colombia", https://doi.org/10.7910/DVN/YGO4JF, Harvard Dataverse, V1, UNF:6:jLcmChA/Y6UTy + 3J7bLqxg =  = [fileUNF].

## Abbreviations

ACEP: American College of Emergency Physicians
FAST: Focused Assessment for Sonographic Trauma
POCUS: Point-of-care ultrasound
