# Supplementary material for: Evaluation of remote radiologist-interpreted point-of-care ultrasound for suspected dengue patients in a primary health care facility in Colombia
Source: Infect Dis Poverty. 2023 Sep 28;12:90. doi: 10.1186/s40249-023-01141-9 (PMC10537978; doi:10.1186/s40249-023-01141-9)
Supplement: Supplementary file 1 — Additional file 1: Table. American College of Emergency Physicians (ACEP) suggested ultrasound image quality rating. [file 40249_2023_1141_MOESM1_ESM.pdf]

Table. American College of Emergency Physicians (ACEP) suggested ultrasound image quality rating.

Grading	Definition
1	No recognizable structures, no objective data can be gathered
2	Minimally recognizable structures but insufficient for diagnosis
3	Minimal criteria met for diagnosis, recognizable structures but with some technical or other flaws
4	Minimal criteria met for diagnosis, all structures imaged well, and diagnosis easily supported
5	Minimal criteria met for diagnosis, all structures imaged with excellent image quality and diagnosis completely supported
