# Supplementary material for: Evaluation of remote radiologist-interpreted point-of-care ultrasound for suspected dengue patients in a primary health care facility in Colombia
Source: Infect Dis Poverty. 2023 Sep 28;12:90. doi: 10.1186/s40249-023-01141-9 (PMC10537978; doi:10.1186/s40249-023-01141-9)
Supplement: Supplementary file 2 — Additional file 2: Table. Results of point-of-care ultrasound image quality by age, sex, and dengue clinical classification of patients. [file 40249_2023_1141_MOESM2_ESM.pdf]

Table. Results of point-of-care ultrasound image quality by age, sex, and dengue clinical classification of patients

Ultrasound image	Quality score	Total	Age in years					Sex			Dengue classification		
			≤ 18	19–29	30–59	≥ 60	*P*	Female	Male	*P*	Dengue without warning signs	Dengue with warning signs	*P*
Right lung apex	1 and 2	51	24	9	14	4	0.03	28	23	0.9	28	23	0.09
	3 and 4	126	78	28	14	6		70	56		52	74	
Right lung base	1 and 2	39	22	5	8	4	0.2	28	11	0.02	21	18	0.2
	3 and 4	138	80	32	20	6		71	67		59	79	
Right posterolateral alveolar	1 and 2	132	80	26	20	6	0.6	75	57	0.5	56	76	0.2
	3 and 4	45	23	10	8	4		23	22		24	21	
Hepatorenal space	1 and 2	137	77	27	24	9	0.3	72	65	0.2	64	73	0.2
	3 and 4	39	26	9	3	1		25	14		14	25	
Left lung apex	1 and 2	45	26	6	10	3	0.3	28	17	0.3	18	27	0.4
	3 and 4	133	77	31	18	7		71	62		62	71	
Left lung base	1 and 2	47	30	7	8	2	0.6	28	19	0.5	26	21	0.1
	3 and 4	130	72	30	20	8		71	59		54	76	
Left posterolateral alveolar	1 and 2	154	87	31	28	8	0.1	84	70	0.5	71	83	0.5
	3 and 4	23	15	6	0	2		14	9		9	14	
Splenorenal space	1 and 2	151	84	32	27	8	0.3	81	70	0.1	72	79	0.1
	3 and 4	25	17	5	1	2		17	8		8	17	
Pericardial space	1 and 2	130	70	33	21	6	0.06	73	57	0.8	56	74	0.4
	3 and 4	48	33	4	7	4		26	22		24	24	
Gallbladder appearance	1 and 2	55	32	9	12	2	0.3	32	23	0.7	29	26	0.2
	3 and 4	121	70	28	15	8		67	54		51	70	
Gallbladder wall	1 and 2	96	57	21	11	7	0.4	44	52	0.004	45	51	0.3
	3 and 4	77	46	16	13	2		52	25		31	46	
Pelvic space	1 and 2	31	16	8	5	2	0.8	22	9	0.05	14	17	0.9
	3 and 4	147	87	29	23	8		77	70		66	81	
